# Supplementary material for: Effects of supplementing a polyphenol-rich sugarcane extract through drinking water on egg production and quality of laying hens
Source: PLoS One. 2025 Feb 5;20(2):e0317292. doi: 10.1371/journal.pone.0317292 (PMC11798506; doi:10.1371/journal.pone.0317292)
Supplement: Attachment — Submitted filename: PONE-D-24-45645_corrected.pdf [file pone.0317292.s001.pdf]

PLOS ONE

Effects of supplementing a polyphenol-rich sugarcane extract through drinking water on egg production and quality of laying hens

--Manuscript Draft--

Manuscript Number:	PONE-D-24-45645
Article Type:	Research Article
Full Title:	Effects of supplementing a polyphenol-rich sugarcane extract through drinking water on egg production and quality of laying hens
Short Title:	Sugarcane polyphenol and laying hen performance
Corresponding Author:	Namalika Darshani Karunaratne, BVSc; MSc; PhD<br>University of Peradeniya Faculty of Veterinary Medicine and Animal Science<br>Peradeniya, SRI LANKA
Keywords:	Antioxidant; Poultry; layer production; egg mass; yolk colour; albumen height
Abstract:	Polyphenols are a wide group of naturally occurring compounds found in plants and have the potential to safeguard living cells. The objective was to evaluate whether the inclusion of a polyphenol-rich sugarcane extract (PRSE) in drinking water could improve egg production and the quality of commercial layers. A total of 120 Shaver Brown hens, aged 43 weeks, were randomly allocated to 12 litter-floor pens in two open-sided poultry houses. The pens were divided into two treatment groups: one receiving 0% (control) and the other 0.05% PRSE in drinking water throughout the study duration. The treatments were prepared by adding PRSE manually into the drinking water daily, and water was given ad libitum. The birds were given commercial layer feed throughout the study. The number of eggs produced, abnormal eggs, and mortality were recorded daily. Egg weight, yolk colour, yolk height, albumen height, Haugh units, and antioxidant properties, were measured at weeks 45, 47 and 49. Supplementing PRSE in the drinking water did not impact hen-day egg production, hen-housed egg production, egg weight, egg mass, or feed conversion ratio. However, there was a trend toward significance in egg weight at week 45 (P = 0.06). The results indicated that PRSE supplementation led to a significant reduction in yolk colour during week 45 (P = 0.001), although no differences were observed in subsequent weeks. Yolk height, thick albumen height, and haugh units were unaffected by the treatment, while thin albumen height showed a trend towards reduction in the PRSE group at weeks 47 and 49 (P = 0.05). The DPPH assay revealed a significant increase in antioxidant capacity in the PRSE group at week 45 (P = 0.02). The 0.05% PRSE supplementation in drinking water has a limited impact on egg production and quality, with only temporary effects on antioxidant capacity.
Order of Authors:	Namalika Darshani Karunaratne, BVSc; MSc; PhD
	Sasmitha De Silva
	Minoli Darshani Herath
	Ruvini Liyanage, BSc, MSc, PhD
	Pabodha Weththasinghe, BSc, PhD
	Barana Jayawardhana, BSc, PhD
	Eranga De Seram
	Anil Pushpakumara, BSc, PhD
	Mathew Flavel, BVSc
Additional Information:	
Question	Response
Financial Disclosure<br><br>Enter a financial disclosure statement that	The author(s) received no specific funding for this work.

Powered by Editorial Manager® and ProduXion Manager® from Aries Systems Corporation

describes the sources of funding for the work included in this submission. Review the submission guidelines for detailed requirements. View published research articles from *PLOS ONE* for specific examples.<br><br>This statement is required for submission and **will appear in the published article** if the submission is accepted. Please make sure it is accurate.<br><br>**Funded studies**<br>Enter a statement with the following details:<br>• Initials of the authors who received each award<br>• Grant numbers awarded to each author<br>• The full name of each funder<br>• URL of each funder website<br>• Did the sponsors or funders play any role in the study design, data collection and analysis, decision to publish, or preparation of the manuscript?<br><br>Did you receive funding for this work?	
**Competing Interests**<br><br>Use the instructions below to enter a competing interest statement for this submission. On behalf of all authors, disclose any competing interests that could be perceived to bias this work—acknowledging all financial support and any other relevant financial or non-financial competing interests.<br><br>This statement is required for submission and **will appear in the published article** if the submission is accepted. Please make sure it is accurate and that any funding sources listed in your Funding Information later in the submission form are also declared in your Financial Disclosure statement.<br><br>View published research articles from *PLOS ONE* for specific examples.	Mathew Flavel, a co-author of this manuscript is attached to the company that supplies the polyphenol product. However, he did not involve with manuscript writing, data analysis and interpretation.

**NO authors have competing interests**<br><br>Enter: *The authors have declared that no competing interests exist.*<br><br>**Authors with competing interests**<br><br>Enter competing interest details beginning with this statement:<br><br>*I have read the journal's policy and the authors of this manuscript have the following competing interests: [insert competing interests here]*<br><br>* typeset	
**Ethics Statement**<br><br>Enter an ethics statement for this submission. This statement is required if the study involved:<br><br>• Human participants<br>• Human specimens or tissue<br>• Vertebrate animals or cephalopods<br>• Vertebrate embryos or tissues<br>• Field research<br><br>Write "N/A" if the submission does not require an ethics statement.<br><br>General guidance is provided below. Consult the submission guidelines for detailed instructions. **Make sure that all information entered here is included in the Methods section of the manuscript.**	The experimental protocol received approval from the Ethics Committee of the Faculty of Veterinary Medicine and Animal Science at the University of Peradeniya (Ethical Clearance Certificate Number VERC-24-02)

Powered by Editorial Manager® and ProduXion Manager® from Aries Systems Corporation

**Format for specific study types**<br><br>**Human Subject Research (involving human participants and/or tissue)**<br>• Give the name of the institutional review board or ethics committee that approved the study<br>• Include the approval number and/or a statement indicating approval of this research<br>• Indicate the form of consent obtained (written/oral) or the reason that consent was not obtained (e.g. the data were analyzed anonymously)<br><br>**Animal Research (involving vertebrate animals, embryos or tissues)**<br>• Provide the name of the Institutional Animal Care and Use Committee (IACUC) or other relevant ethics board that reviewed the study protocol, and indicate whether they approved this research or granted a formal waiver of ethical approval<br>• Include an approval number if one was obtained<br>• If the study involved *non-human primates*, add *additional details* about animal welfare and steps taken to ameliorate suffering<br>• If anesthesia, euthanasia, or any kind of animal sacrifice is part of the study, include briefly which substances and/or methods were applied<br><br>**Field Research**<br><br>Include the following details if this study involves the collection of plant, animal, or other materials from a natural setting:<br>• Field permit number<br>• Name of the institution or relevant body that granted permission	
**Data Availability**<br><br>Authors are required to make all data underlying the findings described fully available, without restriction, and from the time of publication. PLOS allows rare exceptions to address legal and ethical concerns. See the PLOS Data Policy and FAQ for detailed information.	Yes - all data are fully available without restriction

A Data Availability Statement describing where the data can be found is required at submission. Your answers to this question constitute the Data Availability Statement and **will be published in the article**, if accepted.<br><br>**Important:** Stating 'data available on request from the author' is not sufficient. If your data are only available upon request, select 'No' for the first question and explain your exceptional situation in the text box.<br><br>Do the authors confirm that all data underlying the findings described in their manuscript are fully available without restriction?	
**Describe where the data may be found in full sentences. If you are copying our sample text, replace any instances of XXX with the appropriate details.**<br><br>• If the data are **held or will be held in a public repository**, include URLs, accession numbers or DOIs. If this information will only be available after acceptance, indicate this by ticking the box below. For example: *All XXX files are available from the XXX database (accession number(s) XXX, XXX.).*<br>• If the data are all contained **within the manuscript and/or Supporting Information files**, enter the following: *All relevant data are within the manuscript and its Supporting Information files.*<br>• If neither of these applies but you are able to provide **details of access elsewhere**, with or without limitations, please do so. For example:<br><br>*Data cannot be shared publicly because of [XXX]. Data are available from the XXX Institutional Data Access / Ethics Committee (contact via XXX) for researchers who meet the criteria for access to confidential data.*<br><br>*The data underlying the results presented in the study are available from (include the name of the third party*	All relevant data are within the manuscript and its Supporting Information files

and contact information or URL).<br>• This text is appropriate if the data are owned by a third party and authors do not have permission to share the data.<br><br>* typeset	
Additional data availability information:	Tick here if the URLs/accession numbers/DOIs will be available only after acceptance of the manuscript for publication so that we can ensure their inclusion before publication.

Manuscript Click here to access/download;Manuscript;Manuscript.docx

Sugarcane polyphenol and laying hen performance

Effects of supplementing a polyphenol-rich sugarcane extract through drinking water on egg production and quality of laying hens

Namalika D. Karunaratne[1]*, Sasmitha De Silva[1], Minoli Herath[1], Ruvini Liyanage[2], Pabodha Weththasinghe[3], Barana C. Jayawardana[3], Eranga De Seram[1], Anil Pushpakumara[1], Mathew Flavel[4]

[1] Department of Farm Animal Production and Health, Faculty of Veterinary Medicine and Animal Science, University of Peradeniya, Sri Lanka

[2] National Institute of Fundamental Studies, Kandy, Sri Lanka

[3] Department of Animal Science, Faculty of Agriculture, University of Peradeniya, Sri Lanka

[4] Bioactives Division, The Product Makers, Melbourne VIC, Australia

*Corresponding author

Email: nkarunaratne@vet.pdn.ac.lk (NDK)

Abstract

Polyphenols are a wide group of naturally occurring compounds found in plants and have the potential to safeguard living cells. The objective was to evaluate whether the inclusion of a polyphenol-rich sugarcane extract (PRSE) in drinking water could improve egg production and the quality of commercial layers. A total of 120 Shaver Brown hens, aged 43 weeks, were randomly allocated to 12 litter-floor pens in two open-sided poultry houses. The pens were divided into two treatment groups: one receiving 0% (control) and the other 0.05% PRSE in drinking water throughout the study duration. The treatments were prepared by adding PRSE manually into the drinking water daily, and water was given *ad libitum*. The birds were given commercial layer feed throughout the study. The number of eggs produced, abnormal eggs, and mortality were recorded daily. Egg weight, yolk colour, yolk height, albumen height, Haugh units, and antioxidant properties, were measured at weeks 45, 47 and 49. Supplementing PRSE in the drinking water did not impact hen-day egg production, hen-housed egg production, egg weight, egg mass, or feed conversion ratio. However, there was a trend toward significance in egg weight at week 45 (P = 0.06). The results indicated that PRSE supplementation led to a significant reduction in yolk colour during week 45 (P = 0.001), although no differences were observed in subsequent weeks. Yolk height, thick albumen height, and haugh units were unaffected by the treatment, while thin albumen height showed a trend towards reduction in the PRSE group at weeks 47 and 49 (P = 0.05). The DPPH assay revealed a significant increase in antioxidant capacity in the PRSE group at week 45 (P = 0.02). The 0.05% PRSE supplementation in drinking water has a limited impact on egg production and quality, with only temporary effects on antioxidant capacity.

Keywords: antioxidant, poultry, layer production, egg mass, yolk colour, albumen height

Introduction

The poultry industry is increasingly challenged by oxidative stress, which arises from various factors such as mycotoxins and heat stress, leading to lipid peroxidation and cellular apoptosis [1]. This oxidative stress is detrimental to poultry health, negatively impacting performance and liveability by suppressing the immune system [2]. Consequently, mitigating oxidative stress through the incorporation of antioxidants, particularly natural ones, has become a focal point in enhancing poultry health and productivity [3].

In recent years, the integration of natural bioactive compounds into poultry diets, especially for laying hens, has garnered substantial attention within the field of poultry nutrition. Among these bioactive compounds, polyphenols, which are naturally occurring substances found in plants are of particular interest due to their wide range of biological activities, including antioxidant, anti-inflammatory, anticancer, and antimicrobial properties [4,5]. Sugarcane (*Saccharum officinarum*), a perennial grass commonly found in South Asia, Southeast Asia, and other tropical and subtropical regions, is notably rich in polyphenolic compounds, such as phenolic acids, glycosides, flavonoids, and fatty acids [6,7].

These polyphenolic compounds have been shown to positively influence various physiological processes in poultry, contributing to improved gut health, enhanced growth performance, reduced lipid peroxidation, and the mitigation of oxidative stress. Moreover, polyphenols are integral to plant defense mechanisms, and when incorporated into livestock diets, they offer numerous health benefits [8,9]. Notably, phenolic compounds derived from plant materials, such as sugarcane extracts, have demonstrated anticoccidial effects in poultry [10].

Therefore, understanding the composition and properties of polyphenols in sugarcane is essential for advancing agricultural practices and animal-derived food production.

Despite the extensive body of knowledge regarding the role of polyphenols in livestock nutrition, there remains a significant research gap concerning the specific impact of polyphenol-rich sugarcane extract (PRSE) on egg production and quality in laying hens. While previous studies have examined polyphenols from various plant sources, the unique composition of sugarcane-derived polyphenols and their potential effects on layer production have not been thoroughly investigated. Moreover, the limited studies that are available on this topic have yielded inconsistent results, emphasizing the need for further research [11].

In response to this research gap, the present study was designed to assess the impact of PRSE supplementation in drinking water on the egg production and quality of laying hens. It was hypothesized that PRSE administration in drinking water would enhance both egg production and quality in laying hens. This investigation aims to provide a more comprehensive understanding of the effects of polyphenol-rich sugarcane extracts on poultry performance, thereby contributing valuable insights to the field of poultry nutrition.

Materials and methods

The experimental protocol received approval from the Ethics Committee of the Faculty of Veterinary Medicine and Animal Science at the University of Peradeniya, as indicated by the Ethical Clearance Certificate Number VERC-24-02.

Experimental treatments

Two sets of treatments were formulated by manually blending 0% and 0.05% of PRSE in drinking water individually. Water was provided as *ad libitum* throughout the duration of the study.

Birds and housing

A total of 120 Shaver Brown laying hens, aged 43 weeks, were randomly allocated to 12 floor pens, with 10 birds per pen. Each pen contained paddy husk evenly spread across the concrete floor. These pens were located in two open-sided poultry houses (6.2 m in length, 6 m in width, 2.2 m in height) at the Udaperadeniya Livestock Farm, affiliated with the Faculty of Agriculture, University of Peradeniya, Sri Lanka. Each poultry house was divided into six pens (1.8 m in length, 1.8 m in width, 1.7 m in height). The two treatments were randomly assigned to three pens in each house, resulting in six replications per treatment. Each pen was equipped with a manual plastic pan feeder (10 kg capacity; 40 cm pan diameter) and a manual plastic bell drinker (6 l capacity; 23 cm diameter). Additionally, each pen contained three nest boxes (30.5 cm in length, 30.5 cm in width, 28 cm in height). The laying hens were provided with commercial layer feed in mash form throughout the study period. Each pen received 1.2 kg of feed per day, corresponding to 120 g of feed per bird per day. The environmental and management conditions were maintained according to the recommended standards of the Shaver Brown management guidelines.

Study material

In this study, a recently identified sugarcane extract was employed, which was initially developed using the hydrophobic resin method described by [5]. The extract, available in a liquid-to-paste form and exhibiting a brown coloration, was produced by The Product Makers Pvt Ltd in Melbourne, Australia. The sugarcane extract contained a total polyphenol content of

221 mg/g and a flavonoid content of 53.8 mg/g, and the pH of a 1% solution at 20ºC was measured at 3.74 [12]. Furthermore, the extract demonstrated a total antioxidant activity of 18,837 µmol/g. Elemental analysis showed concentrations of sodium (Na), potassium (K), calcium (Ca), magnesium (Mg), and zinc (Zn) at 170, 130, 6600, 3000, and 15 mg/kg, respectively, with selenium (Se) and chromium (Cr) present at 0.3 and 2.4 mg/kg [5].

Data collection

The total number of eggs produced and the number of abnormal eggs (including broken, cracked shell, soft shell, abnormal shaped, and double-yolked eggs) were recorded daily for each pen throughout the study period. Hen-day egg production and hen-housed egg production were calculated by dividing the total number of eggs per pen by the number of hen-days and hens housed, respectively [13]. Eggs laid in each pen over a 24-hour period were collected and individually weighed using a digital scale (SF-400, LK) at 45, 47 and 49 weeks of age, allowing for the calculation of average egg weight per pen on these days. Egg mass was determined by multiplying the total number of eggs by the average egg weight on the specified days [14]. Mortality was recorded daily, and the feed conversion ratio was calculated biweekly (at 45, 47 and 49 weeks of age) as g of feed consumed per g of egg mass [13]. Humane endpoints during the research were determined based on several criteria, including significant impairment of the bird's mobility that affects its capacity to access food and water, substantial weight loss, and the presence of a severe defect with no possibility of recovery.

Determination of egg quality

Two of the collected eggs from each pen at 45, 47, and 49 weeks of age were used to analyze thin albumen height, thick albumen height, yolk height, and yolk colour. Immediately after collection, eggs were transported to a laboratory and analyzed the same day. Thick and thin albumin height, yolk height, and yolk color values used in this study were the mean of three measurements obtained directly from each egg after breaking it on a glass surface. Eggs were measured with an electronic scale (SF-400, LK) before breaking the egg. The heights of the thick and thin albumin and yolk were measured using a digital Haugh meter (Model S6428, b.c. Ames Co., Waltham, Mass, USA), and a yolk colour fan (ROCHE, Yolk Colour fan, 1155, Switzerland) was used to measure yolk colour. Three readings from different places were taken and the mean value was used for the calculations.

Haugh Unit (HU) was calculated according to the formula below.

$$HU = 100*\log_{10}(h-1.7w^{0.37}+7.56)$$

Observed mean height of the thick albumen in millimetres (h) and mean value of the weight of eggs per pen in grams (w) were used for calculations [15].

Determination of antioxidant properties of eggs

Five eggs collected from the same pen were carefully cracked, and the yolks were separated using an egg yolk separator. The yolks from all five eggs were pooled together and thoroughly mixed using a hand mixer. A 60 ml aliquot of the pooled egg yolk mixture was then freeze-dried using a freeze dryer (SUPERMODULYO-230, Milford, MA 01757) and stored at -20°C until further analysis. The resulting freeze-dried egg yolk powder was utilized for subsequent sample extraction. Powdered egg yolk samples (2 g) were extracted into 20 ml of 80% methanol (analytical grade) by magnetic stirring for 30 minutes. Then the content was

centrifuged for 15 minutes at 6000 rpm at 4°C using a centrifuge (5340R, Germany) and the supernatant was separated and stored at -20°C until analysis.

Determination of 2,2-Diphenyl-1-Picrylhydrazyl Assay (DPPH) Radical Scavenging Capacity

The procedure described by [16] was used with minor modifications. A hundred microliters of DPPH solution were added to different extract volumes (0–150 l). The reaction mixture was allowed to stand in the dark for 30 min at room temperature, and the absorbance was measured at 517 nm. The results were expressed in mmol/dm^3 Trolox equivalents per gram dry weight.

Determination of Ferric Reducing Antioxidant Powder (FRAP) Assay

The FRAP reagent (150μl) containing 2,4,6-Tripyridyl-S-triazine (TPTZ) (10 mM in10 mM HCl), $FeCl_3$ (10 mM), and 30 µl of pH 3.6 acetate buffer (300 mM) with the ratio of 1:1:10 (v/v/v) was pre-incubated at 37°C for 8 min. The sample extract (50 µl) was added to 150 µl of the FRAP reagent and incubated for 30 min at room temperature. The absorbance was measured at 593 nm, and results were expressed in µmoldm^{-3} Fe^{2+} equivalents per gram dry weight.

Statistical analysis

The study employed a randomized complete block design, utilizing a broiler room as a block to alleviate environmental impacts. Egg production and quality, and antioxidant properties were analyzed through a two-sample T-test using the Proc mixed model in the Stata statistical software. The significance level was set at $P \leq 0.05$. Before variance analysis, data normality was assessed through the Shapiro-Wilk test.

Results

Egg production

Supplementation of PRSE in drinking water revealed no significant impact on egg production, egg weight, egg mass and feed conversion ratio of laying hens across each evaluated period or when considering the total duration of the study as detailed in Table 1 and Table 2. The egg weight at week 2 tended to be higher in PRSE supplemented group compared to the control (P = 0.06).

Table 1. Effect of supplementing polyphenol-rich sugarcane extract (PRSE) in drinking water on the egg production of laying hens

**Variable**	**PRSE [1] concentration (%) in drinking water**		**SEM[2]**	***P* value**
	**0**	**0.05**		
Weekly hen-day egg production (%)				
44 wk of age	91.4	88.8	2.696	0.35
45 wk of age	94.3	94.3	2.857	1.00
46 wk of age	91.9	95.5	3.280	0.30
47 wk of age	89.5	93.1	5.124	0.49
48 wk of age	93.4	91.7	2.591	0.51
49 wk of age	95.8	92.9	3.216	0.37
Bi-weekly hen-day egg production (%)				
43 to 45 wk of age	92.9	91.5	2.558	0.62
45 to 47 wk of age	90.7	94.3	3.946	0.39
47 to 49 wk of age	94.6	92.3	2.506	0.37
Total hen-day egg production (%)	92.7	92.7	2.397	0.99
Weekly hen-housed egg production (%)				
44 wk of age	91.4	88.8	2.696	0.35
45 wk of age	94.3	94.3	2.857	1.00
46 wk of age	91.9	95.5	3.280	0.30
47 wk of age	88.8	93.1	5.628	0.46
48 wk of age	91.9	91.7	3.280	0.94
49 wk of age	94.3	92.9	3.762	0.71
Bi-weekly hen-housed egg production (%)				
43 to 45 wk of age	92.9	91.5	2.558	0.62

45 to 47 wk of age	90.4	94.3	4.196	0.37
47 to 49 wk of age	93.1	92.3	3.195	0.80
Total hen-housed egg production (%)	92.1	92.7	2.746	0.83

[a-b]Means within a row without a common superscript differ significantly ($P < 0.05$).
[1]PRSE - Polyphenol rich sugarcane extract.
[2]SEM – Pooled standard error of the mean (n=6).

Table 2. Effect of supplementing polyphenol-rich sugarcane extract (PRSE) in drinking water on the production performance of laying hens

**Variable**	**PRSE[1] concentration (%) in drinking water**		**SEM[2]**	***P* value**
	**0**	**0.05**		
Egg weight (g)				
45 wk of age	59.8	61.5	0.831	0.06
47 wk of age	60.4	61.1	1.005	0.48
49 wk of age	61.6	61.2	0.938	0.68
Egg mass (g/hen/day)				
45 wk of age	56.7	58.5	4.228	0.67
47 wk of age	53.1	54.1	3.353	0.76
49 wk of age	51.2	54.1	5.120	0.59
Feed conversion				
45 wk of age	2.15	2.08	0.155	0.67
47 wk of age	2.28	2.25	0.155	0.89
49 wk of age	2.48	2.23	0.322	0.45

[a-b]Means within a row without a common superscript differ significantly ($P < 0.05$).
[1]PRSE - Polyphenol rich sugarcane extract.
[2]SEM – Pooled standard error of the mean (n=6).

Egg quality

At 45 weeks, egg yolk color was reduced with PRSE supplementation compared to the control group. However, no significant differences in yolk color were observed at 47 and 49 weeks of age between the treatments. PRSE supplementation led to a reduction in thin albumen height at the evaluated time points, except at 45 weeks. No significant treatment effects were observed on yolk height, thick albumen height, or Haugh units across all weeks of age analyzed.

Table 3. Effect of supplementing polyphenol-rich sugarcane extract (PRSE) in drinking water on egg quality

Variable	PRSE[1] concentration (%) in drinking water		SEM[2]	*P* value
	0	0.05		
Yolk colour				
45 wk of age	7.3	6.6	0.120	0.001
47 wk of age	6.7	6.7	0.112	1.00
49 wk of age	6.3	6.7	0.151	0.29
Yolk height (mm)				
45 wk of age	17.4	17.5	0.537	0.87
47 wk of age	17.8	18.0	0.256	0.87
49 wk of age	18.4	18.8	0.161	0.24
Thick albumen height (mm)				
45 wk of age	7.7	7.6	0.206	0.96
47 wk of age	8.0	8.0	0.341	0.99
49 wk of age	8.4	8.7	0.196	0.47
Thin albumen height (mm)				
45 wk of age	2.0	1.9	0.104	0.66
47 wk of age	2.6	2.0	0.171	0.05
49 wk of age	3.1	2.7	0.111	0.05
Haugh units				
45 wk of age	87.3	87.0	1.237	0.14
47 wk of age	89.2	88.9	1.786	0.62
49 wk of age	91.0	92.7	1.000	0.71

[a-b]Means within a row without a common superscript differ significantly ($P < 0.05$).
[1]PRSE - Polyphenol rich sugarcane extract.
[2]SEM – Pooled standard error of the mean (n=6).

Antioxidant properties

Supplementation of PRSE in drinking water reduced the 2,2-diphenyl-1-picrylhydrazyl (DPPH) assay value, indicating an enhanced free radical scavenging capacity in laying hens at 45 weeks of age (Table 2). However, supplementation of PRSE in drinking water demonstrated no

significant effect on reducing ferric ion to ferrous ion in laying hens across each evaluation period (P > 0.05), as detailed in Table 2.

Table 4. Effect of supplementing polyphenol-rich sugarcane extract in drinking water on the antioxidant properties of egg yolk

Variable	PRSE[1] concentration (%) in drinking water		SEM[2]	*P* value
	0	0.05		
2,2-diphenyl-1-picrylhydrazyl assay (mMTrolox Eq/g)				
Week 45	72.2	45.6	6.152	0.02
Week 47	51.7	51.6	1.767	0.98
Week 49	47.7	62.7	4.561	0.10
Ferric Reducing Antioxidant Power Assay (mMFe^{2+} Eq/g)				
Week 45	436052.0	425965.3	46671.39	0.92
Week 47	430418.7	417682.0	29532.87	0.84
Week 49	506055.3	474492.0	54280.62	0.79

[a-b]Means within a row without a common superscript differ significantly ($P < 0.05$).
[1]PRSE - Polyphenol rich sugarcane extract.
[2]SEM – Pooled standard error of the mean (n=6).

Discussion

This study evaluated the effects of supplementing polyphenol-rich sugarcane extract (PRSE) in the drinking water of Shaver Brown laying hens, focusing on its impact on internal egg quality, antioxidant capacity, and production performance. The findings offer insights into how PRSE supplementation influences egg quality and antioxidant properties, while also highlighting the complexity of these interactions and the need for further research.

A significant observation was the reduction in yolk color at week 45 in the PRSE-supplemented group. This reduction is likely due to the oxidation of pigments such as xanthophylls and carotenoids, leading to a lighter yolk color. Such an outcome is undesirable as

it suggests a potential decrease in the nutritional quality of the eggs and deviates from the deep yellow color preferred by consumers [17]. The return of yolk color to normal levels in subsequent weeks might indicate an initial disruption in the hens' antioxidant defense mechanisms due to PRSE introduction, followed by a gradual adaptation to the supplementation. The transient nature of this effect raises questions about the role of PRSE in modulating oxidative processes within the egg yolk, potentially reducing the stability or bioavailability of yolk pigments [18].

Despite the negative impact on yolk color, other internal egg quality parameters, including yolk height, thick albumen height, and Haugh units, were not significantly affected by PRSE supplementation, indicating that the overall internal quality of the eggs remained largely intact [19]. However, the reduction in thin albumen height observed at weeks 47 and 49 suggests that PRSE may have subtle effects on egg white consistency over time, warranting further investigation.

The antioxidant capacity of the egg yolk was also assessed, revealing a significant reduction in DPPH radical-scavenging activity at week 45 in the PRSE group. A lower DPPH value indicates an enhanced antioxidant capacity, suggesting that the polyphenols in PRSE were initially effective in improving the eggs' ability to neutralize free radicals [5]. However, this effect was not sustained over time, implying that the antioxidant benefits of PRSE may be short-lived or that the hens' metabolism adapted to the supplementation. This outcome could be due to the specific nature of the polyphenols in PRSE or an insufficient dosage.

In contrast to previous studies that reported significant effects of polyphenol supplementation on egg antioxidant properties, this study's results may have been influenced by the approach used to assess antioxidant capacity. Previous research often employed different

methodologies, such as measuring plasma and yolk malonaldehyde concentrations, GSH activity in plasma and yolk, and shelf life, which may provide more accurate insights into antioxidant properties [20,21]. Future studies should consider incorporating these parameters to obtain a more comprehensive understanding of polyphenol's effects on egg quality.

The supplementation of 0.05% PRSE in the drinking water did not lead to statistically significant improvements in hen-day egg production, hen-housed egg production, egg mass, or feed conversion ratio throughout the study period. Although there was a slight indication of significance in egg weight during week 2 ($P = 0.06$), these findings suggest that the concentration and method of PRSE administration used in this experiment may not have been sufficient to elicit notable benefits under the specific conditions tested.

Polyphenols are widely recognized for their antioxidant, anti-inflammatory, antibacterial, anticancer, and antistress properties [22,23]. Previous studies have demonstrated their potential to improve health and productivity in laying hens, including reduced oxidative stress, enhanced egg quality, improved eggshell integrity, and positive effects on gut health [22,24,25]. However, the lack of significant effects observed in this study could be attributed to several factors.

First, the concentration of PRSE used (0.05%) may have been too low to produce measurable benefits. The efficacy of polyphenols is often dose-dependent [26], with higher concentrations sometimes required to observe significant outcomes. Conversely, excessively high doses of polyphenols can reduce nutrient digestibility and negatively affect weight gain [11], highlighting the importance of determining an optimal dosage. Additionally, the method of administering polyphenols through drinking water rather than feed could have influenced their bioavailability and absorption, potentially diminishing their effectiveness. Polyphenols might

interact differently with other dietary components when administered in water, affecting their impact.

Another consideration is the specific polyphenol profile of the sugarcane extract used in this study. Most previous studies that observed significant effects on egg production, quality, and antioxidant properties utilized polyphenols derived from other plant sources, such as tea, grape pomace, and other plant varieties [22,27-29]. The polyphenol content and type of polyphenols can vary significantly between these different sources, making it challenging to compare the results of this study with those of others. Some research has even found reduced egg quality parameters, including eggshell weight, eggshell strength, and albumen quality, with polyphenol supplementation derived from plant sources like tea polyphenols. These variations emphasize the complexity of polyphenol effects on laying hens and suggest that the specific polyphenols in sugarcane extract may interact differently with the metabolism of birds.

Furthermore, the age of the hens may play a crucial role in their response to polyphenol supplementation. Most studies that reported positive effects of polyphenols on egg quality and antioxidant properties involved a late laying period, that is above 50 weeks [20,22,27,28]. In contrast, studies that found negative or non-significant effects often involved an early laying period [30,31]. The age-related differences in metabolism and absorption of polyphenols could influence egg quality outcomes, suggesting that older hens might benefit more from polyphenol supplementation than younger ones. The age of the hens at the start of this study (43 weeks) places them in a mature phase of production, where their physiological responses to dietary interventions might differ from those in early and late laying periods. This factor should be considered in future studies to optimize polyphenol supplementation strategies. Further, the six-week duration of this study might not have been long enough to observe the full effects of PRSE

supplementation. Some benefits of polyphenols may take longer to manifest and extending the study duration could provide more comprehensive insights into the long-term impact of PRSE on laying hens. Furthermore, strain-specific responses to polyphenol supplementation should also be considered, as different strains of commercial layers might exhibit varying sensitivities to dietary changes.

Given the mixed results of polyphenol supplementation reported in previous studies, it is essential to continue investigating the effects of PRSE and other polyphenol sources in laying hens. Future research should explore varying concentrations of PRSE, alternative administration methods, and longer study durations. Additionally, examining the effects of polyphenol supplementation across different strains and at various stages of the laying cycle could provide valuable insights into optimizing egg production and quality through dietary interventions.

Acknowledgments

Acknowledgment is granted to the staff of the Udaperadeniya Livestock Farm, Faculty of Agriculture of the University of Peradeniya for their valuable support in the execution of the animal trials.

References

1. Oke OE, Akosile OA, Oni AI, Opowoye IO, Ishola CA, Adebiyi JO, et al. Oxidative stress in poultry production. Poult Sci. 2024;103: 104003.

2. Awad WA, Ghareeb K, Dadak A, Hess M, Böhm J. Single and combined effects of deoxynivalenol mycotoxin and a microbial feed additive on lymphocyte DNA damage and oxidative stress in broiler chickens. PLoS One. 2014;9: e88028.

3. Birben E, Sahiner UM, Sackesen C, Erzurum S, Kalayci O. Oxidative stress and antioxidant defense. World Allergy Organ J. 2012;5: 9–19.

4. Awais MM, Akhtar M. Evaluation of some sugarcane (*Saccharum officinarum* L.) extracts for immunostimulatory and growth promoting effects in industrial broiler chickens. Pak Vet J. 2012;32: 398-402.

5. Ji J, Yang X, Flavel M, Shields ZPI, Kitchen B. Antioxidant and anti-diabetic functions of a polyphenol-rich sugarcane extract. J Am Coll Nutr. 2019;38: 670–80.

6. Singh A, Lal UR, Mukhtar HM, Singh PS, Shah G, Dhawan RK. Phytochemical profile of sugarcane and its potential health aspects. Pharmacogn Rev. 2015;9: 45–54.

7. Feehan J, Prakash MD, Stojanovska L, Flavel MR, Kitchen B, Apostolopoulos V. Immunomodulatory properties of polyphenol-rich sugarcane extract on human monocytes. Biologics. 2021;1: 211–21.

8. Abdel-Moneim AME, Shehata AM, Alzahrani SO, Shafi ME, Mesalam NM, Taha AE, et al. The role of polyphenols in poultry nutrition. J Anim Physiol Anim Nutr. 2020;104: 1851–66.

9. Iqbal Y, Cottrell JJ, Suleria HAR, Dunshea FR. Gut microbiota-polyphenol interactions in chicken: A review. Animals. 2020;10: 1391-1409.

10. Akhtar M, Hafeez MA, Muhammad F, Haq AU, Anwar MI. Immunomodulatory and protective effects of sugar cane juice in chickens against Eimeria infection. 2008;32: 463-7.

11. Abd El-Hack ME, Salem HM, Khafaga AF, Soliman SM, El-Saadony MT. Impacts of polyphenols on laying hens' productivity and egg quality: A review. J Anim Physiol Anim Nutr. 2023;107: 928–47.

12. Ji J, Flavel M, Yang X, Chen OCY, Downey L, Stough C, et al. A polyphenol rich sugarcane extract as a modulator for inflammation and neurological disorders. PharmaNutrition. 2020;12: 100187.

13. Herwig E, Walk CL, Bedford M, Schwean-Lardner K, Classen HL. Contrasting the effects of phytase and pure myo-inositol on the performance, digestibility, blood and egg yolk inositol levels and digestion physiology of laying hens. Br Poult Sci. 2021;62: 517-27.

14. Herwig E, Abbott D, Schwean-Lardner KV, Classen HL. Assessing the effect of rate and extent of starch digestion on laying hen performance. Poult Sci. 2019;98: 2940–7.

15. Jin YH, Lee KT, Lee WI, Han YK. Effects of storage temperature and time on the quality of eggs from laying hens at peak production. Asian Australas J Anim Sci. 2010;24: 279–84.

16. Sanjeevkumar CB, Londonkar RL, Kattegouda UM, Tukappa NKA. Screening of in vitro antioxidant activity of chloroform extracts of *Bryonopsis laciniosa* fruits. IJCMAS. 2016;5: 590-597.

17. Gouveia L, Veloso V, Reis A, Fernandes H, Novais J, Empis J. *Chlorella vulgarisused* to colour egg yolk. J Sci Food Agri. 1996;70: 167–72.

18. Flachowsky G. Natural antioxidants in avian nutrition and reproduction. Anim Feed Sci Technol. 2003;103: 177–8.

19. Williams KC. Some factors affecting albumen quality with particular reference to haugh unit score. World's Poult Sci J. 1992;48: 5–16.

20. Simitzis P, Spanou D, Glastra N, Goliomytis M. Impact of dietary quercetin on laying hen performance, egg quality and yolk oxidative stability. Anim Feed Sci Technol. 2018;239: 27–32.

21. Xie T, Bai SP, Zhang KY, Ding XM, Wang JP, Zeng QF, et al. Effects of *Lonicera confusa* and Astragali Radix extracts supplementation on egg production performance, egg quality, sensory evaluation, and antioxidative parameters of laying hens during the late laying period. Poult Sci. 2019;98: 4838–47.

22. Wang X cui, Wang X hong, Wang J, Wang H, Zhang H jun, Wu S geng, et al. Dietary tea polyphenol supplementation improved egg production performance, albumen quality, and magnum morphology of Hy-Line Brown hens during the late laying period1. J Anim Sci. 2018;96: 225–35.

23. Cueva C, Silva M, Pinillos I, Bartolomé B, Moreno-Arribas MV. Interplay between dietary polyphenols and oral and gut microbiota in the development of colorectal cancer. Nutrients. 2020;12: 625.

24. Uuganbayar D, Bae IH, Choi KS, Shin IS, Firman JD, Yang CJ. Effects of green tea powder on laying performance and egg quality in laying hens. Asian Australas J Anim Sci. 2005;18: 1769–74.

25. Laudadio V, Ceci E, Lastella NMB, Tufarelli V. Dietary high-polyphenols extra-virgin olive oil is effective in reducing cholesterol content in eggs. Lipids in Health and Disease. 2015;14: 5.

26. Nunes KC, Eyng C, Pintro PTM, Garcia RG, Murakami AE, Vital ACP, et al. Dietary inclusion of dehydrated bocaiuva pulp increases the antioxidant potential of quail eggs. J Anim Physiol Anim Nutr. 2019;103: 64–71.

27. Kara K, Kocaoğlu Güçlü B, Baytok E, Şentürk M. Effects of grape pomace supplementation to laying hen diet on performance, egg quality, egg lipid peroxidation and some biochemical parameters. J Appl Anim Res. 2016;44: 303–10.

28. Yuan ZH, Zhang KY, Ding XM, Luo YH, Bai SP, Zeng QF, et al. Effect of tea polyphenols on production performance, egg quality, and hepatic antioxidant status of laying hens in vanadium-containing diets. Poult Sci. 2016;95: 1709–17.

29. Romero C, Arija I, Viveros A, Chamorro S. Productive performance, egg quality and yolk lipid oxidation in laying hens fed diets including grape pomace or grape extract. Animals. 2022;12: 1076-92.

30. Liu HN, Liu Y, Hu LL, Suo YL, Zhang L, Jin F, et al. Effects of dietary supplementation of quercetin on performance, egg quality, cecal microflora populations, and antioxidant status in laying hens. Poult Sci. 2014;93: 347–53.

31. Zhu YF, Wang JP, Ding XM, Bai SP, Qi SRN, Zeng QF, et al. Effect of different tea polyphenol products on egg production performance, egg quality and antioxidative status of laying hens. Anim Feed Sci Technol. 2020;267: 114544.

Click here to access/download
Supporting Information
Data (1).xlsx
