# Supplementary material for: Effects of supplementing a polyphenol-rich sugarcane extract through drinking water on egg production and quality of laying hens
Source: PLoS One. 2025 Feb 5;20(2):e0317292. doi: 10.1371/journal.pone.0317292 (PMC11798506; doi:10.1371/journal.pone.0317292)
Supplement: Attachment — Submitted filename: Review for PLoS ONE.docx [file pone.0317292.s002.docx]

Dear Editor of PLOS ONE

I have completed my review of manuscript PONE-D-24-45645, titled "Effects of supplementing a polyphenol-rich sugarcane extract through drinking water on egg production and quality of laying hens." During my evaluation, I identified several areas that require improvement in both the methodology and data presentation. Specifically, the abstract and introduction should be revised for better readability and to provide a more thorough explanation of how PRSE affects egg production and internal egg quality. Some sections need additional clarification, particularly regarding the mechanisms behind the reduction in yolk color and the duration of the antioxidant effects. Additionally, presenting the data in a more comprehensive manner, such as incorporating graphs and providing further details on experimental conditions, would enhance the manuscript. Overall, I believe that with the appropriate revisions, this manuscript has the potential to make a meaningful contribution to the field, but it will require a “major revision” before it can be considered for acceptance.

Kind regards,

Aan Andri Yano

**Comments to authors:**

I have reviewed your manuscript and identified several areas for improvement. The introduction would benefit from a clearer structure and more background on the poultry industry, polyphenols, and sugarcane. Additionally, more explanation is needed regarding the mechanisms by which PRSE affects yolk color and antioxidant capacity. Presenting the results graphically and providing more details on treatment duration, environmental conditions, and the rationale for the chosen PRSE dose would also improve the manuscript.

Abstract

The abstract provides a thorough overview of the study's design, methodology, and findings, but a few elements could enhance clarity and completeness, especially considering the title:

1. Please consider improving the overall flow of the abstract to enhance readability.
2. Kindly include a clear statement of the hypothesis or specific aims related to egg production and quality to strengthen the introduction (first sentence).
3. It would be helpful to summarize the actual numbers or percentages related to egg production or quality in the abstract for greater detail.
4. Including the significance level when the p-value is 0.06 may be unnecessary and could lead to confusion (line 39)
5. The conclusion should be highlighted more prominently.

Introduction

Overall: The introduction lacks a clear problem statement and relevant background literature, resulting in a disjointed flow that diminishes readability. Additionally, the novelty of the study is not sufficiently articulated, and transitions between topics need improvement to provide a more cohesive and informative narrative.

1. The flow of the introduction could be improved, as it currently lacks coherence and may not be enjoyable to read.
2. The first paragraph should serve as an introduction to the poultry industry rather than jumping directly into the problem (line 51-56).
3. The connection between the discussion of polyphenols and sugarcane lacks fluidity; please consider enhancing this linkage (line 57-64).
4. Include the specific polyphenol content in sugarcane, along with its advantages, and compare it to other types of grasses (line 65).
5. Although the benefits of sugarcane are mentioned, the study primarily examines its extract; please provide clarification on the findings from previous research regarding the impact of sugarcane extract on livestock productivity (line 70).
6. Furthermore, please elaborate on the effectiveness of polyphenols in enhancing livestock productivity and indicate the specific levels at which productivity improvements have been observed in previous studies (line 65-69).

Materials and Methods:

Overall: The methods section provided is fairly comprehensive, but several aspects could be enhanced or clarified to better align with the title. Here are some suggestions

1. Kindly provide a more detailed breakdown of the basal diet’s nutrient content (e.g., protein, fat, fiber), particularly regarding its interaction with the polyphenol extract.
2. The method for selecting eggs for quality analysis could be more detailed. For example, were specific days chosen for sampling based on production cycles, or was it random? (Line 136).
3. While the housing conditions are described, details about environmental factors (e.g., temperature, humidity) during the study could be important, as these can impact egg production and quality. (line 95)
4. Please include the total length of the treatment period to help contextualize the results.

Results

The data should be presented in graphical form, as the authors have shown weekly performance trends that are better illustrated this way. Also, there are no different superscripts where the means within a row differ significantly. This should be added.

Discussion

Overall: Based on the excerpt you've provided from the manuscript, it appears the study thoroughly examined the effects of polyphenol-rich sugarcane extract (PRSE) on the egg production, internal egg quality, and antioxidant capacity in laying hens. Here is a detailed review highlighting the potential weaknesses, and areas for further clarification or investigation.

Reduction in Yolk Color:

Line 219 🡪 The study notes a reduction in yolk color at week 45, which is attributed to the oxidation of pigments such as xanthophylls and carotenoids. However, there is no detailed explanation about the potential mechanisms of how PRSE may have caused this effect. It would be helpful to discuss why polyphenols may have led to oxidation or reduced stability of carotenoids in the yolk.

To clarify: Did the authors measure specific carotenoid concentrations in the yolk to confirm the reduction? Additionally, how do the antioxidant effects of PRSE relate to the decrease in yolk color, given that antioxidants are typically expected to protect against oxidation?

Antioxidant Effect:

Line 235 🡪 The study reports a significant reduction in antioxidant capacity at week 45, followed by a loss of this effect over time. The manuscript mentions that the antioxidant benefits of PRSE may be short-lived or that the hens' metabolism adapted to the supplementation.

To clarify: Was the PRSE supplementation able to sustain its effect on antioxidant capacity over the entire study period? It would be useful to explore whether the dosage or bioavailability of PRSE might have influenced the duration of antioxidant effects.

Lack of Significant Effects on Egg Production:

Line 249 🡪 The lack of significant effects on egg production, mass, feed conversion ratio, and egg weight are major findings, which suggests that the concentration or method of PRSE supplementation used might not have been effective under the study conditions.

To clarify: The authors suggest that the low concentration of PRSE (0.05%) might have been insufficient. Were higher concentrations tested in preliminary studies or are there literature reports suggesting optimal dosages for polyphenol supplementation? This could help to better contextualize the choice of concentration.

Line 269 🡪 The authors mention that the lack of significant effects in their study contrasts with previous studies that reported positive effects of polyphenols on egg quality and production. This difference is partly attributed to the methodology used to assess antioxidant capacity (e.g., DPPH vs. other markers like GSH or malondialdehyde).

To clarify: It would be helpful for the authors to more thoroughly discuss the methodological differences that might account for these discrepancies. How does the DPPH method compare with other antioxidant assays in terms of sensitivity to polyphenol effects? Moreover, could the polyphenol content of the sugarcane extract vary widely between batches, which might account for inconsistencies in results?

Age of the Hens

Line 279 🡪 The age of the hens (43 weeks) is highlighted as a possible factor influencing the outcomes, with older hens (typically above 50 weeks) showing more pronounced benefits from polyphenol supplementation in previous studies.

Potential Clarification: The authors suggest that hens at 43 weeks may be in a mature phase of production, but do not explain why the age-specific response to polyphenol supplementation could differ so markedly. More evidence or references to studies on age-related polyphenol metabolism in poultry would strengthen this argument.

Conclusion

One limitation of the current study is the lack of exploration into the optimal dosages and administration methods of PRSE. Future research should also consider the variability in responses across different strains and laying cycle stages
