# Supplementary material for: Effects of supplementing a polyphenol-rich sugarcane extract through drinking water on egg production and quality of laying hens
Source: PLoS One. 2025 Feb 5;20(2):e0317292. doi: 10.1371/journal.pone.0317292 (PMC11798506; doi:10.1371/journal.pone.0317292)
Supplement: Attachment — Submitted filename: Review for revised manuscript.docx [file pone.0317292.s003.docx]

Review for revised manuscript

Abstract

The abstract in the revised manuscript provides a clear summary of the study’s aim, methodology, and findings, but could benefit from improved flow and additional details:

1. The mention of a p-value of 0.06 could cause confusion and may be omitted (Line 39), please revise.
2. The conclusion should be more prominent and clearer in its emphasis (Line 45).

Introduction

The introduction in the revised manuscript is informative but lacks a smooth flow between topics:

1. The connection between polyphenols and sugarcane should be explained more clearly (Line 57-64).
2. More specific information about the polyphenol content in sugarcane and its comparison to other grasses would strengthen the background (Line 65), please revise.
3. The study should provide clearer explanations of previous research findings on sugarcane extract and its effects on livestock (Line 70). Please add them.

Materials and Methods

The methods section in the revised manuscript is comprehensive but could benefit from more detail in the following areas:

1. The method for selecting eggs for quality analysis could be clarified, particularly whether specific days were chosen for sampling based on production cycles (Line 136).
2. Environmental conditions, such as temperature and humidity, should be specified as they may influence egg production and quality (Line 95).

Results

The data in the revised manuscript would be clearer if presented graphically:

1. Trends in performance would be better illustrated in graphical form (Line 45-49).
2. Superscripts should be used in tables to indicate significant differences within rows (Table 2).

Discussion

The discussion in the revised manuscript addresses key findings but could be improved in several areas:

1. The reduction in yolk color at week 45 should be further explained with a discussion on the potential mechanisms behind it, particularly how PRSE may have caused oxidation or reduced stability of carotenoids (Line 219).
2. The loss of antioxidant effects over time should be discussed in more detail, particularly regarding whether the PRSE supplementation was able to sustain its antioxidant effects throughout the study period (Line 235).
3. The age of the hens as a factor influencing the outcomes should be explored in more depth, with more references or evidence regarding age-specific responses to polyphenol supplementation (Line 279).

Conclusion

The conclusion in the revised manuscript is a valuable summary of the study’s findings, but it could be enhanced by:

1. Emphasizing the need for future research to focus on optimizing PRSE dosages, administration methods, and considering age-specific responses in poultry (Line 279).
