# Supplementary material for: Effects of supplementing a polyphenol-rich sugarcane extract through drinking water on egg production and quality of laying hens
Source: PLoS One. 2025 Feb 5;20(2):e0317292. doi: 10.1371/journal.pone.0317292 (PMC11798506; doi:10.1371/journal.pone.0317292)
Supplement: Attachment — Submitted filename: Response to Reviewers.docx [file pone.0317292.s004.docx]

The authors would like to extend their gratitude to the editor and all three reviewers for their valuable comments and suggestions to improve the quality of the manuscript.

Reviewer 2

| Reviewer comment | Response to reviewer |
| --- | --- |
| The mention of a p-value of 0.06 could cause confusion and may be omitted (Line 39), please revise. | P value was removed (L 39). |
| The conclusion should be more prominent and clearer in its emphasis (Line 45). | The conclusion statement of the abstract was revised as suggested (L 45-46). |
| The connection between polyphenols and sugarcane should be explained more clearly (Line 57-64). | A statement was added to make the connection between polyphenols and sugarcane (L 75-76). |
| More specific information about the polyphenol content in sugarcane and its comparison to other grasses would strengthen the background (Line 65), please revise. | Specific information about sugarcane (compared to other crops) was added as background information (L 73-74). |
| The study should provide clearer explanations of previous research findings on sugarcane extract and its effects on livestock (Line 70). Please add them. | Explanation related to sugarcane extract effects on livestock, especially poultry was added to the introduction (L 83-84). |
| The method for selecting eggs for quality analysis could be clarified, particularly whether specific days were chosen for sampling based on production cycles (Line 136). | The study lasted for six weeks, with egg collection conducted every two weeks, resulting in three sampling days for egg quality analysis. Previous nutritional studies on egg quality, typically lasting around 20 weeks, have collected samples at four-week intervals. However, given the shorter duration of this trial, samples were collected more frequently at two-week intervals. The sampling schedule was also influenced by budgetary constraints, which limited the number of samples that could be analyzed. |
| Environmental conditions, such as temperature and humidity, should be specified as they may influence egg production and quality (Line 95). | The experiment was carried out in an open-sided poultry house, and therefore, the temperature and humidity could not be controlled. Further, temperature and humidity were different throughout the day, and also throughout the trial duration, and the authors could not monitor those parameters continuously since it is not an environmentally controlled house. However, the average temperature and humidity in that region were reported in the manuscript (L 123-125). |
| Trends in performance would be better illustrated in graphical form (Line 45-49). | Performance data were included as 2 graphs in the results section (Fig 1 and Fig 2; L 207-213). |
| Superscripts should be used in tables to indicate significant differences within rows (Table 2). | Superscripts were added to tables to indicate significant differences within rows (Table 3 and Table 4). |
| The reduction in yolk color at week 45 should be further explained with a discussion on the potential mechanisms behind it, particularly how PRSE may have caused oxidation or reduced stability of carotenoids (Line 219). | Potential mechanisms that can lead to the reduction of yolk colour were added to the discussion (L 255-261). |
| The loss of antioxidant effects over time should be discussed in more detail, particularly regarding whether the PRSE supplementation was able to sustain its antioxidant effects throughout the study period (Line 235). | This was discussed in more detail as suggested by the reviewer (L 289-294). |
| The age of the hens as a factor influencing the outcomes should be explored in more depth, with more references or evidence regarding age-specific responses to polyphenol supplementation (Line 279). | Explanations related to age-specific responses were added to the discussion (L 361-366). |
| The conclusion in the revised manuscript is a valuable summary of the study’s findings, but it could be enhanced by: Emphasizing the need for future research to focus on optimizing PRSE dosages, administration methods, and considering age-specific responses in poultry (Line 279). | The conclusion statement was revised by including the suggested points (L 394). |

Editor

| Editor’s comment | Response to editor |
| --- | --- |
| According to authors' statement, Mathew Flavel should not have been listed as co-author as he/she did not involve in the manuscript preparation and writing and did not intellectually contribute to the manuscript. Please review the authorship requirement of the journal for more details. | The authors intended to convey that Mathew Flavel did not impose his opinions on the study design or outcomes, so the statement was rephrased accordingly in the cover letter.  “The funder provided support in the form of salaries for authors [NDK], but did not inappropriately influence the study design, data collection and analysis, decision to publish, or preparation of the manuscript.” |
| Any changes to the reference list should be mentioned in the rebuttal letter that accompanies your revised manuscript | Two new references were added to the discussion section, and therefore, the reference list was updated (L 516-521).  42. Batal AB, Parsons CM. Effects of age on nutrient digestibility in chicks fed different diets. Poultry Science. 2002; 81(3): 400-407.  43. Del Vesco, AP, Khatlab, AS, Goes ESR, Utsunomiya, KS, Vieira, JS, Oliveira Neto AR, et al. Age-related oxidative stress and antioxidant capacity in heat-stressed broilers. Animal. 2017; 11(10): 1783-1790. |
